# Edge Functionalized Graphene Layers for (Ultra) High Exfoliation in Carbon Papers and Aerogels in the Presence of Chitosan

**DOI:** 10.3390/ma13010039

**Published:** 2019-12-20

**Authors:** Silvia Guerra, Vincenzina Barbera, Alessandra Vitale, Roberta Bongiovanni, Andrea Serafini, Lucia Conzatti, Luigi Brambilla, Maurizio Galimberti

**Affiliations:** 1 Politecnico di Milano, Department of Chemistry, Materials and Chemical Engineering “G. Natta”, Via Mancinelli 7, 20131 Milan, Italy; silvia.guerra@polimi.it (S.G.); andrea.serafini@polimi.it (A.S.);; 2 Politecnico di Torino, Department of Applied Science and Technology, Corso Duca degli Abruzzi 24, 10129 Torino, Italy; alessandra.vitale@polito.it (A.V.); roberta.bongiovanni@polito.it (R.B.); 3 Istituto di Scienze e Tecnologie Chimiche (SCITEC) “Giulio Natta”, Via De Marini 6—16149 Genova, Italy

**Keywords:** few layers graphene, chitosan, aerogel, carbon paper

## Abstract

Ultra-high exfoliation in water of a nanosized graphite (HSAG) was obtained thanks to the synergy between a graphene layer edge functionalized with hydroxy groups and a polymer such as chitosan (CS). The edge functionalization of graphene layers was performed with a serinol derivative containing a pyrrole ring, serinol pyrrole (SP). The adduct between CS and HSAG functionalized with SP was formed simply with a mortar and pestle, then preparing water dispersions stable for months in the presence of acetic acid. Simple casting of such dispersions on a glass support led to carbon papers. Aerogels were prepared through the freeze-dry procedure. Exfoliation was observed in both these families of composites and ultra-high exfoliation was documented in aerogels swollen in water. Carbon papers and aerogels were stable for months in solvents in a wide range of solubility parameter and in a pretty wide range of pH. By considering that a moderately functionalized nanographite was straightforwardly exfoliated in water in the presence of one of the most abundant biobased polymers, the obtained results pave the way for the simple and sustainable preparation of graphene-based nanocomposites. HSAG–SP/CS adducts were characterized by wide angle X-ray diffraction (WAXD), scanning and transmission electron microscopy (SEM, TEM and HRTEM), Fourier transform infrared spectroscopy (FT-IR), X-ray photoelectron spectroscopy (XPS) and Raman spectroscopy. Thermal stability of the composites was studied by thermogravimetric analysis (TGA) and their direct electrical conductivity with the four-point probe method.

## 1. Introduction

In the graphene era, increasing attention is focused on graphene functionalization and graphene-based composites [1,2,3]. Remarkable research has been done on graphene papers and aerogels, as they are suitable for many applications [4,5,6,7,8]. Lightness, flexibility and conductivity together with a preparation method characterized by a low environmental impact are objectives mandatory for such materials. Large amounts of graphene layers are prepared from graphitic materials through the top down approach and outstanding properties are achieved only if the degree of exfoliation is high/very high and the sp^2^ hybridization of the carbon atoms remains substantially unaltered. According to the nomenclature for graphene and graphene related materials [9], besides the single layer of graphene, two stacked graphene layers form a bi-layer graphene and a few layers graphene consists of stacked graphene layers in a number from three to ten. In a recent study [10] on graphene-based commercial products from 60 producers, it may be read that “the majority of the companies are producing less than 10% graphene content and no company is currently producing above 50% graphene content. Most companies produce 4-layer graphene on average”. It is thus evident that the preparation of aerogels and carbon papers based on few-layer graphene is not only challenging from a scientific point-of-view, but it is also of interest for commercial development.

The aim of the present work was the preparation of aerogels and carbon papers based on graphene layers, by using a high surface area nanosized graphite (HSAG) as starting graphitic material. HSAG had a low number of layers stacked in the direction orthogonal to graphene planes and a high shape anisotropy, defined as the ratio between the crystallites size in directions perpendicular and orthogonal to the layers [11]. The most challenging objectives were high/very high exfoliation of the graphitic aggregates, without altering the properties of the graphene layers, and the development of a simple water-based exfoliation process, avoiding the use of organic (aromatic) solvents, which are in most cases not ecofriendly [10,12].

To pursue such objectives, a method was developed based on two main pillars: (i) functionalization of HSAG on the edges, with polar oxygenated groups, to achieve stable dispersions in water, and (ii) use of a bio-polymer with a twofold role: as surfactant to further stabilize the water dispersions and as skeleton of both carbon papers and aerogels.

**Figure 1:**
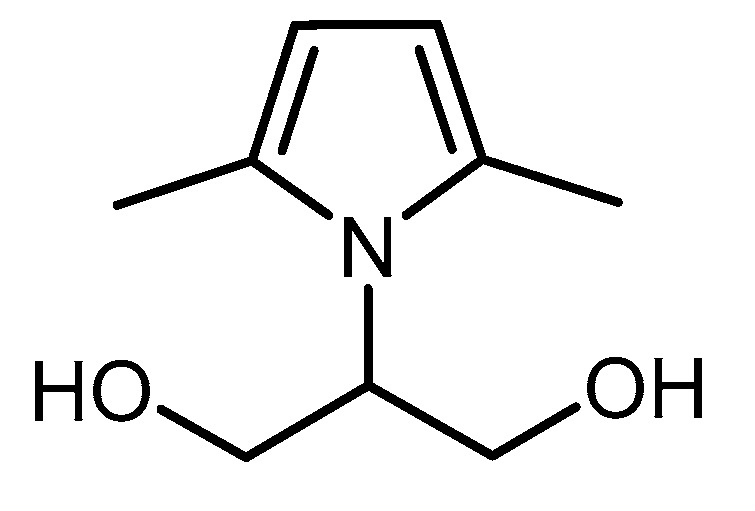
2-(2,5-dimethyl-1*H*-pyrrol-1-yl)-1,3-propanediol (SP).

Functionalization of HSAG was performed with a pyrrole derivative: 2-(2,5-dimethyl-1*H*-pyrrol-1-yl)-1,3- propanediol (serinol pyrrole, SP) [11,13,14,15], whose structure and ^1^H NMR spectrum are reported in Figure 1 and Figure S1, respectively.

SP was obtained from 2-amino 1,3-propandiol with a very high yield and atom efficiency, with water as the co-product of the reaction [11]. The functionalization occurs essentially on the edges of the graphene layers, through a domino reaction [14]. In brief, SP, heated in the presence of the nanographite and air, undergoes oxidation, catalyzed by the carbon material, in benzylic position and an oxidized lateral substituent (typically an aldehyde) is formed. The activated carbon–carbon double bond in PyC gives rise to a cycloaddition reaction on the peripheral positions of the graphene layers. Functionalization can be quantitative as wastes are not formed, and the bulk structure of the graphitic material remains substantially unaltered. HSAG–SP was formed.

The bio-polymer was chitosan (CS), poly (*N*-acetyl-D-glucosamine), a copolymer of [1,4]-linked 2-acetamido-2-deoxy-D-glucopyranose and 2-amino-2-deoxy-D-glucopyranose. This polymer was selected as it can be easily obtained (through deacetylation) from chitin, the second most abundant natural biopolymer in the world. Moreover, chitosan can be dissolved in acidic water and a polycation is formed. A working hypothesis was that the exfoliation of the graphitic aggregates could be favored by the interaction between a polycation, such as chitosan treated with acetic acid, and the aromatic layers. In the scientific literature, the covalent functionalization of graphene sheets with chitosan has been reported [16]: a click coupling reaction was performed between alkynyl-decorated graphene oxide and the graphene sheet. Such functionalization led to “effective exfoliation and enhanced properties of the nanocomposites”.

A novel and simple approach is thus proposed in the present work: the formation of a non-covalent adduct of graphene layers with cationic chitosan is pursued, without the need of troublesome chemical reactions. CS can be easily solubilized in acidic water, preparing a polycation. The adduct between chitosan and the graphitic material is then prepared in water, thanks to the edge functionalization with serinol pyrrole of the graphene layers, whose structure is not appreciably perturbed by the functionalization.

HSAG–SP adducts with CS (HSAG–SP/CS) were prepared by simply mixing HSAG–SP and CS with a mortar and pestle, promoting the formation of a water suspension with the help of acetic acid. Carbon paper and aerogels were prepared starting from stable water suspensions of few-layer graphene. HSAG–SP/CS papers were obtained by casting water suspensions on a glass support and aerogels were prepared by lyophilization of hydrogels. Some of the authors have reported on aerogels and carbon papers containing aggregates of graphene layers [17], prepared by mixing CS and HSAG in water, in the presence of acetic acid. Cation-π interaction was hypothesized at the origin of the stability of HSAG/CS adducts. Pronounced exfoliation was indeed not a robust achievement.

In the present work, chemical composition and thermal stability of the nanocomposites were studied by means of thermogravimetric analysis (TGA). The nature of the functional groups was studied by means of Fourier-transformed infrared spectroscopy (FT-IR) and X-ray photoelectron spectroscopy (XPS). The organization at the solid state and, in particular, the extent of exfoliation, was investigated with wide angle X-ray diffraction (WAXD), scanning electron microscopy (SEM) transmission electron microscopy (TEM) and high-resolution transmission electron microscopy (HRTEM). Raman spectroscopy was used to characterize the HSAG–SP/CS adducts. 

Stability in organic solvents and in aqueous medium of the nanocomposites at different pH was analyzed. Direct electrical conductivity was measured, with the four-probe method. It is worth underlining that samples were prepared with a large amount of HSAG–SP, at least 50% by mass of the final composite, to obtain CS carbon aerogels and papers, characterizing properties such as electrical conductivity, mechanical properties, stability to solvents and pH.

## 2. Materials and Methods 

### 2.1. Materials

Reagents and solvents commercially available were purchased and used without further purification. Chitosan (high purity, Mv 100,000-50,000; degree of acetylation: ≤40 mol%), acetic acid, n-hexane, 2,5-hexanedione and dimethylformamide (DMF) were from Aldrich. Serinol was from Bracco. High surface area graphite (HSAG) with Synthetic Graphite 8427^®^ as trademark was purchased from Asbury Graphite Mills Inc. (Asbury, NY, USA), with a minimum carbon mass of 99.5% and a surface area of 330 m^2^/g.

### 2.2. Synthesis of 2-(2,5-dimethyl-1H-pyrrol-1-yl)-1,3-propanediol (SP)

A mixture of 2,5-hexandione (41.4 g; 0.36 mol) and serinol (30.0 g; 0.33 mol) was poured into a 100 mL round bottomed flask equipped with magnetic stirrer. The mixture was then stirred, at 180 °C, for 3 h in a nitrogen atmosphere. The reaction mixture was distilled under reduced pressure (2 mmHg) at 130 °C and 2-(2,5-dimethyl-1*H*-pyrrol-1-yl)-1,3-propanediol was isolated as a yellow oil (yield: 96%). ^1^H NMR (400MHz, DMSO-*d*6, δ in ppm): 2.16 (s, 6H, –C*H*_3_); 3.63 (m, 2H); 3.76 (m, 2H); 4.10 (quintet, 1H); 4.73 (t, 2H); 5.55 (s, 2H) [11].

### 2.3. Synthesis of the HSAG–SP Adduct 

General procedure: in a 100 mL round bottom flask were put graphite (5 g, 66 mmol) and acetone (15 mL) in sequence. After 15 min sonication with a 2 L ultrasonic bath, a solution of SP (1.116 g, 6.6 mmol) in acetone (5 mL) was added. The resulting instable suspension was further sonicated for 15 min and the solvent was then removed under reduced pressure. The ensuing black powder of HSAG/SP (6.10 g) was poured into a 100 mL round bottomed flask equipped with magnetic stirrer and heated at 180 °C for 3 h. After this time, the mixture was repeatedly washed with acetone (3 × 20 mL) in a Büchner funnel with a sintered glass disc. 5.86 g of black powder (HSAG–SP adduct) were finally obtained [11].

### 2.4. Preparation of Chitosan-Based Nanocomposites

#### 2.4.1. Water Suspensions of HSAG–SP/CS

HSAG–SP adduct (0.4 g) and chitosan (0.4 g) were mixed for 5 min in a mortar with the help of a pestle and then dispersed in water (8 mL). Four drops of an aqueous solution of acetic acid 99.7% (0.010 g) were added and the ensuing suspension was then sonicated for 30 min, using a 2 L ultrasonic bath 260 W (SONICA, SOLTEC Srl, Milan, Italy).

Stable suspensions were obtained using different ratios of chitosan and HSAG-SP (1:1, 1:2, 1:4, 1:6).

#### 2.4.2. Chitosan-Based Carbon Aerogel

The suspensions obtained as reported in Section 2.4.1 were cooled to −30 °C and then lyophilized with a MODULYO EF4-1596 lyophilizer (Edwards, west Sussex, England) under the following experimental conditions: T = −50 °C, P = 5 mbar, lyophilization time t = 24 h. HSAG–SP/CS aerogels with mass ratios equal to 1:1, 4:1 and 1:4 were obtained. The volumetric mass density was determined by measuring the weight of the aerogel sample, by placing it in a cylinder with known volume, then by adding, to fill the volume, small beads with known total mass and packing density. The aerogel volume was estimated by subtracting the volume of the beads. The density of the HSAG–SP/CS aerogel with a 1:1 as mass ratio was found to be about 0.026 g cm^−3^.

#### 2.4.3. Chitosan-Based Carbon Paper 

HSAG–SP/CS suspensions, obtained as reported in Section 2.4.1, were casted on a glass plate (length 12, height 0.5 and width 0.5 cm) in which an adhesive tape (Scotch^®^ Super-Hold Tape—Scotch™ Brand) was used to delimit the area. Sheets were formed after water evaporation, at room temperature and at atmospheric pressure (24 h). HSAG–SP/CS paper thicknesses were 50, 45 and 52 µm for 1:1, 4:1 and 1:4 HSAG–SP and CS mass ratios, respectively.

HSAG–SP/CS carbon paper with a 1:1 as mass ratio showed an area of 12.5 cm^2^ and a bulk density of about 0.81 g cm^−3^.

### 2.5. Characterization of HSAG–SP/CS Composites

#### 2.5.1. Thermogravimetric Analysis (TGA)

TGA tests were performed under flowing N_2_ (60 mL/min) with a TGA SDTA/851 (Mettler Toledo, Columbus, OH, USA) instrument according to the standard method ISO9924-1. Samples (10 mg) were heated from 30 °C to 300 °C at 10 °C/min, kept at 300 °C for 10 min, and then heated up to 550 °C at 20 °C/min. After being maintained at 550 °C for 15 min, they were further heated up to 900 °C and kept at 900 °C for 30 min under flowing air (60 mL/min). Measurements were also performed under flowing air, for temperatures higher than 800 °C.

#### 2.5.2. Elemental Analysis 

Elemental analysis was performed with a Thermo Flash EA 1112 Series CHNS-O analyser, after pre-treating samples in an oven at 100 °C for 12 h.

#### 2.5.3. FT-IR Spectroscopy

FT-IR spectra were recorded between 450 and 4000 cm^−1^ by using a FT-IR Spectrum One (Perkin Elmer, Waltham, MA, USA) equipped with a Universal ATR Sampling Accessory with a diamond crystal.

#### 2.5.4. Wide Angle X-ray Diffraction

Wide-angle X-ray diffraction patterns were obtained in reflection with an automatic D8 Advance diffractometer (Bruker, Billerica, MA, USA), with nickel filtered Cu–K_α_ radiation. Patterns were recorded in 4°–80° as the 2θ range, being 2θ the peak diffraction angle. Distance between crystallographic planes of HSAG was calculated from the Bragg law. The *D_hkℓ_* correlation length, in the direction perpendicular to the hkl crystal graphitic planes, was determined applying the Scherrer equation:

*D_hkℓ_* = *Kλ*/(*β_hkℓ_*cos*θ_hkℓ_*), (1)

where K is the Scherrer constant, λ is the wavelength of the irradiating beam (1.5419 Å, Cu-Kα), *β_hkℓ_* is the width at half height and *θ_hkℓ_* is the diffraction angle. The instrumental broadening, *b*, was determined by obtaining a WAXD pattern of a standard silicon powder 325 mesh (99%), under the same experimental conditions. The width at half height, *β_hkℓ_ =* (*B_hkℓ_* − *b*), was corrected for each observed reflection with *β_hkℓ_* < 1°, by subtracting the instrumental broadening of the closest silicon reflection from the experimental width at half height, *B_hkℓ_*. 

#### 2.5.5. High Resolution X-ray Photoelectron Spectroscopy 

A PHI 5000 VersaProbe instrument (Physical Electronics, Chanhassen, MN, USA) was used for survey, scan and high resolution XPS. The powder was dried in oven at 100 °C for 24 h at atmospheric pressure before analysis and thereafter placed in the XPS pre-chamber overnight, in order to avoid anomalous outgassing during the XPS characterization, performed in UHV conditions (10^−8^ Pa). A monochromatic Al K-alpha X-ray source (1486.6 eV energy, 15 kV voltage and 1 mA anode current, and a power of 25.2 W were used for analysis. Different pass energy values were employed: 187.85 eV for survey spectra and 23.5 eV for high resolution peaks. Analyses were carried out with a take-off angle of 45° and with a 100 μm diameter X-ray spot size on a square area of 1400 × 1400 μm^2^, with the aim to have a good average and better statistics of powder behavior. A double beam (electron and argon ion gun) neutralization system, dedicated to reduce the charging effect on samples, was also employed during data acquisition. All binding energies (BE) were referenced to the C1s line at 284.8 eV. Spectra were analyzed and peak deconvolution was performed using Gauss–Lorentz curves by MultiPak software (version 9.6.0, Physical Electronics, Chanhassen, MN, USA).

#### 2.5.6. Conductivity Measurements 

Direct current (DC) electrical conductivity (s) was measured by the four point probe (FPP) method [18] by using a hand applied FPP device (Jandel Engineering Ltd., Leighton Buzzard, UK) with a probe head with linear arrayed tungsten carbide needles (tip radii 300 mm, needles spacing 635 mm, loads 60 g) coupled with a Keithley 2601 electrometer. Data were acquired and analyzed by CSM/Win Semiconductor Analysis Program software (MDC, Chatsworth, CA, US).

#### 2.5.7. High Resolution Transmission Electron Microscopy (HR-TEM)

HR-TEM investigations on HSAG-SP/CS adducts were carried out with a Philips CM 200 field emission gun microscope operating at 200 kV. Few drops of the water suspensions were deposited on 200 mesh lacey carbon-coated copper grid and air-dried for several hours before analysis. During acquisition of HR-TEM images, performed with low beam current densities and short acquisition times, the samples did not undergo structural transformation. The Gatan Digital Micrograph software was used to estimate in HRTEM micrographs the number of stacked graphene layers and the dimensions of the stacks.

#### 2.5.8. Scanning Electron Microscopy 

The morphology of the external lateral surface of carbon papers (before and after the folding) were characterized with a scanning electron microscope (SEM, Cambridge Stereoscan 360, operating at 20 kV. Before imaging, all the specimens were gold coated using a sputtering system; a layer of approximately 10 nm was deposited.

#### 2.5.9. Raman Spectroscopy 

Raman spectra of samples prepared as powder deposed on glass slide have been recorded with a Labram HR800 Raman spectrometer, Horiba Jobin Yvon, Lille, France coupled to an Olympus BX41 microscop. Experimental parameters: microscope objective 50×, exciting laser line 632.8 nm, laser power 0.5 mW to prevent photo induced degradation of the sample, spectral resolution 2 cm^−1^, and acquisition of four accumulations each of 30 seconds. All e Raman spectra discussed were obtained as the average of four normalized spectra recorded in different points of the sample.

#### 2.5.10. Solvent and pH Resistances

Test was performed placing HSAG–SP/CS carbon paper samples (1 cm^2^, 50 nm thickness for the paper sample) in vials containing 2 mL of water solutions at different pH, hexane and dimethyl formamide (DMF). Vials were left for two months. Acids and bases used to tune pH were HCl (38%), CH_3_COOH (99.85%), NaHCO_3_ and KOH.

## 3. Results and Discussion

### 3.1. Preparation of Adducts of HSAG with Serinol Pyrrole and Chitosan

High surface area graphite (HSAG) was selected as the starting graphitic material. HSAG had a relatively low number of layers stacked in crystalline domains (about 35), a high shape anisotropy (3.1) and about 300 nm as the lateral size of graphitic layers [11]. Chemical composition was then determined by elemental analysis (mass %) performed on samples pre-treated in an oven at 100 °C for 12 h: carbon 99.5, hydrogen 0.4, nitrogen 0.1 and oxygen 0.0. A surface area of 330.3 m^2^/g was determined with BET by applying the ASTM D6556 method.

Functionalization of HSAG with serinol pyrrole was performed as reported in the literature [11,13] and described in the experimental part. Functionalization yield was repeatedly higher than 95%, in line with the literature. 

**Figure 2:**
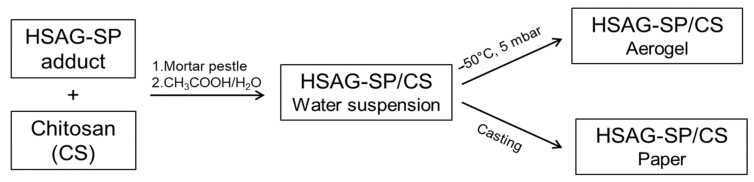
Block diagram describing the preparation of high surface area graphite with serinol pyrrole and chitosan (HSAG–SP/CS) water suspensions, aerogels and papers.

SP is a *Janus* molecule: The pyrrole ring establishes chemical bonds with the graphene layers [11,13,14,15], as explained in theintroduction, and the hydroxy groups easily interact with polar surroundings. Water suspensions of HSAG/SP and chitosan were obtained following the procedure described in the block diagram of Figure 2. 

**Figure 3:**
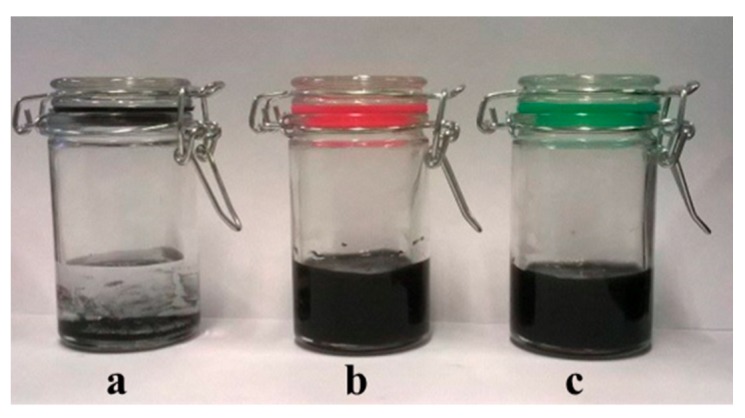
Water suspensions of HSAG (**a**), HSAG-SP/CS after 1 month storage (**b**) and after 30 min centrifugation at 9000 rpm (**c**). HSAG-SP/CS were in a 1:1 mass ratio and the concentration was 1 mg/mL.

As detailed in the Section 2.4, the suspension of HSAG–SP/CS mixtures with different mass ratios (1:1, 2:1, 4:1 and 6:1) were prepared in a water solution of acetic acid and then sonicated for 30 min. Large amounts of suspensions could be prepared being CS and HSAG easily available and the preparation of HSAG–SP very simple. Small samples are shown in Figure 3.

Water suspensions of all the HSAG-SP/CS adducts were observed to be stable for months at rest up to 5 mg/mL as HSAG–SP/CS content. They were stable also after centrifugation. Figure 3 shows water suspensions of HSAG (Figure 3a) and HSAG–SP/CS, with 1:1 as mass ratio and 1 mg/mL as concentration, after 1 month of storage (Figure 3b) and after 30 min centrifugation at 9000 rpm (Figure 3c).

### 3.2. Characterization of Adducts of HSAG with Serinol Pyrrole and Chitosan

**Figure 4:**
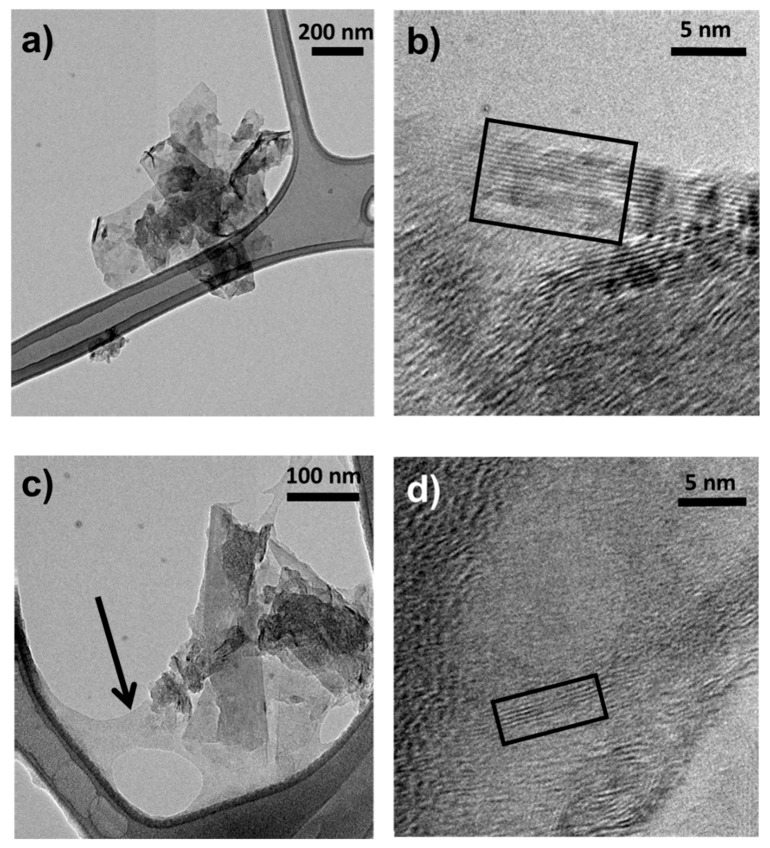
Micrographs of HSAG–SP/CS adducts isolated from supernatant suspensions before (**a**,**b**) and after centrifugation for 15 min at 9000 rpm (**c**,**d**). Micrographs are low magnification bright field TEM (**a**,**c**) and HR-TEM images (**b**,**d**).

The structure of HSAG–SP/CS adduct was investigated by performing TEM and HR-TEM analysis on samples isolated from water suspensions, before and after centrifugation for 30 min at 9000 rpm. In the latter case the supernatant suspension was taken. Figure 4 shows HR-TEM micrographs at lower and higher magnifications.

In the micrographs at lower magnification before (Figure 4a) and after (Figure 4c) sonication, the lateral size of the HSAG–SP/CS adduct is of the same order of magnitude, indicating that the sonication step does not cause appreciable breaking of the graphitic layers. 

By comparing micrographs in Figure 4a,c, it can be seen that CS adheres to the HSAG–SP surface and even to the carbon grid after sonication (the CS layer is indicated by the arrow in Figure 4c). These experimental findings indicate the strong interaction of the carbon materials with the polycation prepared from CS. 

Micrographs at higher magnification (Figure 4b,d) show stacks of graphene layers disposed with a lateral side perpendicular to the beam (indicated in the boxes) from which it is possible to estimate the number of stacked graphene layers [11]. Stacks reported in Figure 4b,d were the most abundant ones in the examined micrographs. As evident in Figure 4b, most of the stacks obtained after sonication are made by about 12–15 graphene layers, a number only slightly lower than the one estimated through WAXD analysis. The centrifuged sample rather reveals stacks of about 4–5 layers (Figure 4d). These findings indicate that the energy given by the sonication process leads to the exfoliation of HSAG stacks and that centrifugation allows to isolate stacks made of few-layer graphene. These results, combined with the evidences from WAXD analysis (reported below in the text), which suggest prevailing disordered stacking of graphene layers, confirm that the combination of cationic CS and turbostratic nanosized graphite functionalized with SP can lead to the preparation of few-layer graphene. It has to be taken into account that only mild sonication was used to promote the exfoliation of the graphite nanoplatelets. It can be reasonably assumed that, by using methods with larger energy, more extended exfoliation could be obtained.

Aerogels and carbon papers were then prepared starting from stable water suspensions. 

### 3.3. Preparation of Aerogels Based on HSAG–SP and Chitosan

**Figure 5:**
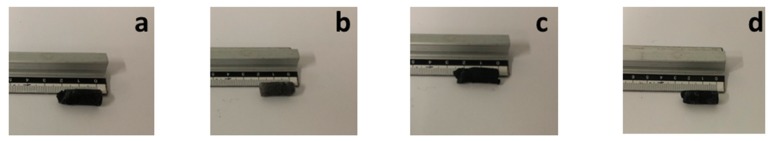
Cylindrical monolithic aerogels made of HSAG–SP/CS. HSAG–SP/CS ratios 1:4 (**a**), 1:1 (**b**), 4:1 (**c**) and 1:1 (**d**).

The procedure for the preparation of aerogels is summarized in Figure 2 and detailed in the experimental part. In brief, the water suspension with the HSAG–SP adduct and CS was frozen to −50 °C and then dried at reduced pressure (5 mbar). Various HSAG–SP/CS mass ratios were used: 1:1, 4:1 and 1:4. Aerogels are shown in Figure 5. 

In all cases monolithic aerogels were obtained. Measured density was in the range from 0.026 to 0.040 g cm^−3^. 

TGA analysis, performed on HSAG–SP, CS and on HSAG–SP/CS aerogel with 1:1 as mass ratio, allowed to estimate the chemical composition and to investigate the thermal stability of the composite materials. Details of the analysis are in the Materials and Methods section and thermographs are shown in Figure S2. HSAG–SP reveals the typical mass loss between 300 °C and 400 °C, due to the decomposition of the organic modifier [11], and no mass loss due to water removal. The curve of CS shows three main decomposition steps: The first occurs in the 50–100 °C range and is reasonably due to the water release (mass loss of about 5%); the further two decompositions between 250 °C and 450 °C are imputable to oxygen and nitrogen containing functional groups, respectively [19]. Curves of HSAG–SP/CS 1:1 aerogel have a shape similar to that of CS with mass losses from 50 °C to 100 °C and from 250 °C to 450 °C. HSAG–SP/CS mass ratios in the aerogels were confirmed by the quantitative evaluation of mass losses in the thermographs. 

### 3.4. Preparation of Carbon Papers Based on HSAG–SP and Chitosan

HSAG–SP/CS papers were prepared as summarized in the block diagram of Figure 2. 

**Figure 6:**
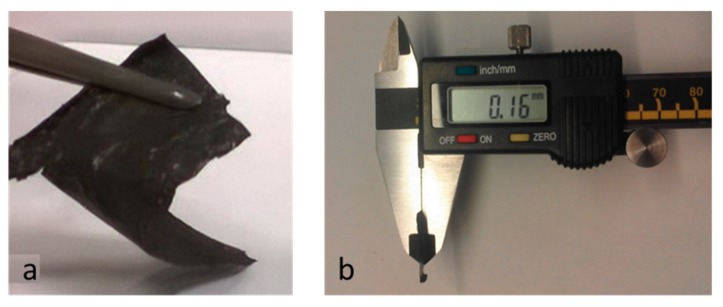
HSAG–SP/CS 1:1 free standing paper (**a**) with 0.16 mm thickness (**b**).

The simple casting of such HSAG–SP/CS water suspensions on a glass support led to the preparation of the carbon papers. Figure 6 shows that a free-standing paper 0.16 mm thick (Figure 6) was obtained by using 1:1 as HSAG–SP/CS ratio. This paper was very flexible, reaching a curvature radius close to zero. The density of the HSAG–SP/CS 1:1 paper was as low as 0.026 g cm^−3^.

### 3.5. Characterization of Adducts, Aerogels and Carbon Papers Based on HSAG–SP and Chitosan

Characterization was performed by means of FT-IR, Raman and XPS spectroscopies, WAXD and SEM analysis, and by measuring the electrical conductivity and investigating the resistance to solvents in a pH range. 

#### 3.5.1. FT-IR Spectroscopy 

**Figure 7:**
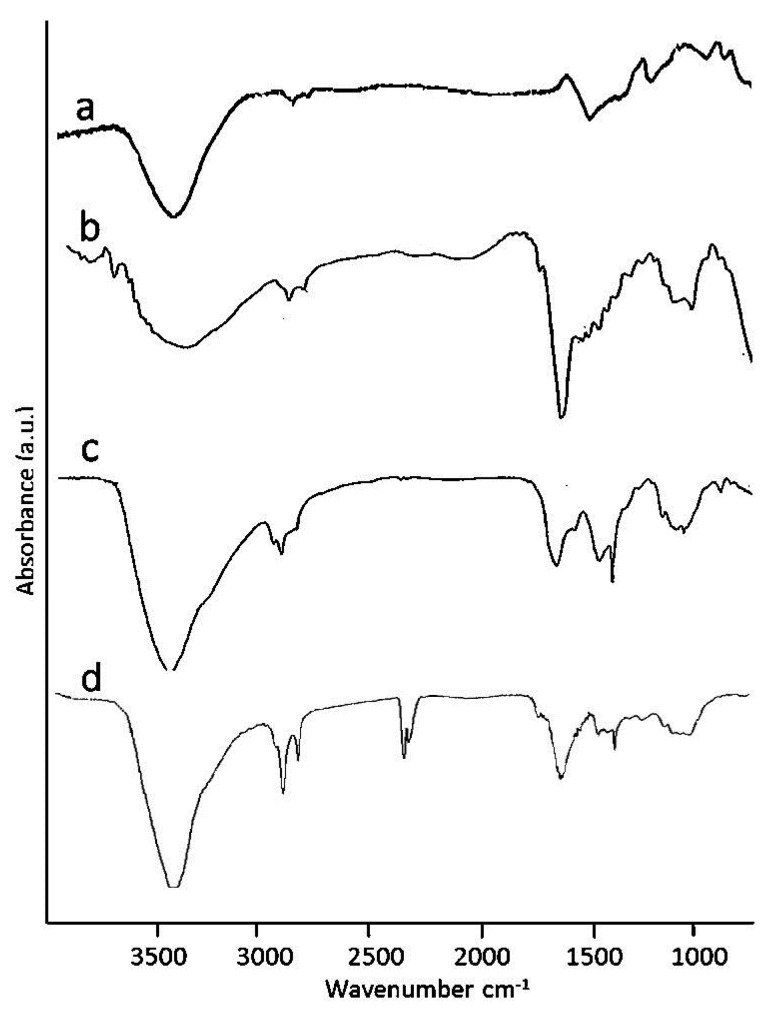
FT-IR spectra of HSAG–SP (**a**), CS (**b**), HSAG–SP/CS 1:1 aerogel (**c**), and HSAG–SP/CS 1:1 aerogel after 1 month storage (**d**).

FT-IR spectroscopy was performed on an HSAG–SP adduct and aerogel. Spectra are in Figure 7.

In Figure 7a, the spectrum of the HSAG–SP adduct is reported. The =C–H stretching of graphene layers is at 2900 cm^−1^. The OH bending of the diol function and the alkenyl bending at visible at 1383 cm^−1^ and 956 cm^−1^ espectively.

The spectrum of pure CS (Figure 7b) shows four peaks located at 3477 cm^−1^, 3444 cm^−1^, 3268 cm^−1^ and 3107 cm^−1^, which are respectively due to the stretching vibrations of –OH, –NH-R and –NH_2_ groups of CS. Bands assigned to CH stretching modes are centred at 2900 cm^−1^. The –C=O stretching vibrations (amide I) of the amide group –C=ONHCH_3_ for crystalline α-chitin are at 1659 cm^−1^ and 1625 cm^−1^ [19]. In the spectrum of HSAG–SP/CS 1:1 aerogel (Figure 7c), there are typical peaks of chitosan (OH and NH stretching) and the HSAG–SP adduct. The FT-IR spectrum of an HSAG–SP/CS aerogel (1:1 ratio) after one month of storage is reported in Figure 7d. It clearly reveals the presence of CO_2_ (Figure 7d), differently for that observed for the freshly prepared sample (Figure 7c). Carbon dioxide (CO_2_) sorption is maybe due to its ultra-low density and to high surface area graphite. 

Samples of HSAG–SP aerogels with different ratios of the components (1:4 and 4:1) were also analyzed. The same type of peak was observed, with relative intensities in line with the relative amount of the composite components. 

#### 3.5.2. Raman Spectroscopy 

**Figure 8:**
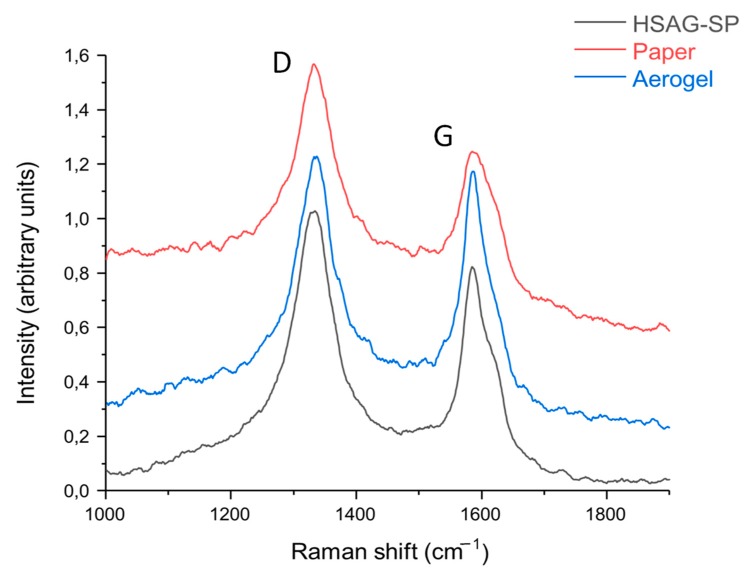
Raman spectra recorded at 632.8 nm of HSAG–SP (**black**), HSAG–SP/CS paper 1:1 (**red**) and HSAG–SP/CS aerogel 1:1 (**blue**). Spectra are shown normalized and stacked for sake of clarity.

Raman spectroscopy was performed on the HSAG–SP adduct, HSAG–SP/CS 1:1 carbon paper and HSAG–SP/CS 1:1 aerogel. Raman spectra reported in Figure 8 have been obtained as average of four normalized spectra recorded in different points of the samples. 

All spectra are dominated by the two scattering lines: the G line at 1582 cm^−1^ and the D line located at the 1335 cm^−1^ characteristic of disordered graphitic materials [20]. The presence of the D line in the spectrum of graphene/graphitic material mechanically treated is ascribed to occurrence of structural disorder due to the reduction of the average dimensions of the stacked graphene layers. The presence of electronically perturbed region close to the edges induces strong confinement effects thus accounting for the appearance in the Raman spectrum of a strong D band [21]. Indeed, the HSAG starting material used in this work was produced through a process of ball milling, which gives very small lateral sized graphitic particles. The presence of the G peak at 1582 cm^−1^ confirms the existence of extended sp^2^ systems in the graphitic particles, which show vibrational properties similar to those of an ideal graphene layer. The Raman spectra of the two samples prepared with HSAG–SP and the CS, namely HSAG–SP/CS 1:1 paper and HSAG-SP/CS 1:1 aerogel, show features of the graphitic moieties with slightly different I_D_/I_G_ ratios with respect to the spectrum of HSAG–SP. No sizable increase of a broad scattering line between the D and the G peaks ascribed to being associated with amorphous sp^3^ carbon structures was observed [22]. Indeed, the increase of I_D_/I_G_ could be due to an increased structural disorder due to the preparation of the sample.

#### 3.5.3. XPS Spectroscopy

**Figure 9:**
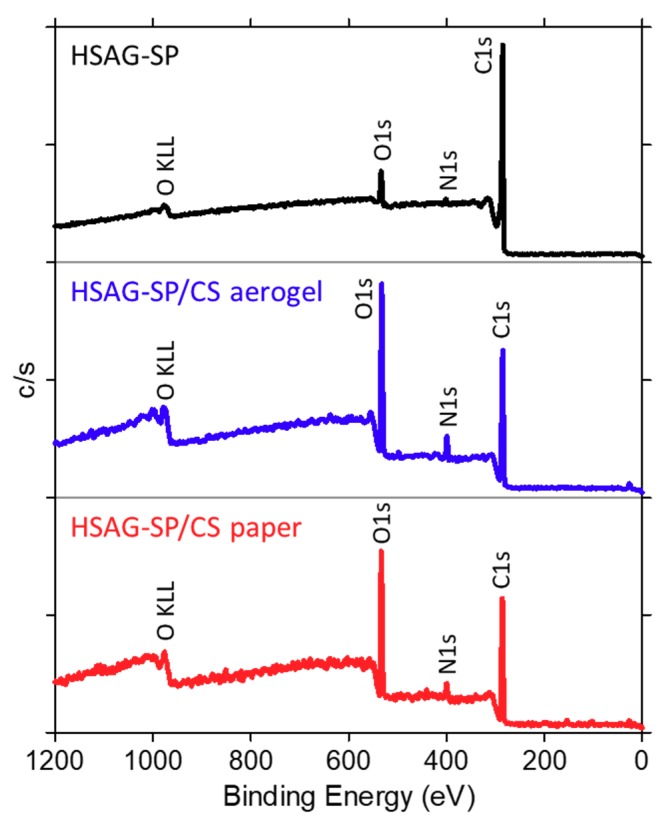
Wide scan XPS spectra of HSAG–SP and of HSAG–SP/CS 1:1 aerogel and paper.

XPS analyses were performed on HSAG–SP and on HSAG–SP/CS 1:1 aerogel and paper. For all samples, wide scan spectra, reported in Figure 9, show three main signals: C1s, O1s and N1s, together with the presence of Si impurities at a BE below 200 eV. Table 1 details the atomic percent concentration of the samples and their relative concentration ratios among the different elements. Both HSAG–SP/CS aerogel and paper present a much higher content of nitrogen, compared to HSAG–SP, indicating that mainly chitosan is being detected during the XPS analysis. Table 1 also reports the theoretical relative amount of atoms in pure chitosan, as calculated taking into consideration the chemical formula [C_6_O_4_H_9_(NH_2_)_x_ (NHCOCH_3_)_y_]_r_, where x and y are fractions of repeating units with free amine and acetylated amine, respectively. The chitosan used in this work has a deacetylation degree of 30%, and therefore x = 0.3 and y = 0.7. It appears that the atomic ratios measured for both HSAG–SP/CS aerogel and paper are very similar to the ones calculated for pure chitosan. Considering that XPS analyses probe only the outer layer of a sample (maximum ca. 100 Å), these results suggest that CS is at the surface of both the aerogel and carbon paper and covers HSAG–SP.

**Figure 10:**
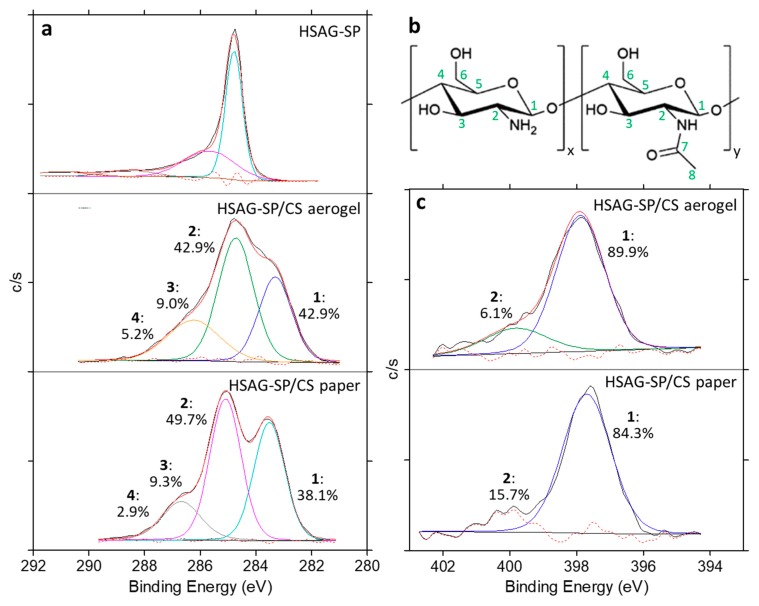
High-resolution C1s XPS spectrum and its deconvolution for HSAG–SP, HSAG–SP/CS 1:1 aerogel and HSAG–SP/CS 1:1 paper (**a**), schematic representation of the chemical structure of partially deacetylated chitosan and labeling of C atoms (**b**), high-resolution N1s XPS spectrum and its deconvolution for HSAG-SP/CS 1:1 aerogel and paper (**c**).

The deconvolution of the high-resolution XPS C1s signal was also performed (Figure 10a). It is evident that the C1s spectrum of both HSAG–SP/CS aerogel and paper is completely different from the C1s envelope of HSAG-SP, while shows the same features of that of chitosan, as reported in the literature [23]. In fact, for pure chitosan four main C1s components are expected (see Figure 10b for carbon atoms labeling): i) atoms C2 and C6 from the glucosamine units, together with carbon atoms C2, C6 and C8 from the *N*-acetylglucosamine rings; ii) C3, C4 and C5 in the glucosamine segment and in the *N*-acetylglucosamine segment; iii) C1 atoms in both segments; iv) C7 in the *N*-acetylglucosamine unit. Taking into account this classification, the relative amount of the C1s components for the chitosan used in this work (i.e., deacetylation degree of 30%) are 35.1%, 44.8%, 14.9% and 5.2%, respectively. These values are very close to the signal area obtained for each C1s contribution for both HSAG–SP/CS aerogel and paper (Figure 10a), thus confirming that XPS analyses mainly detect the chitosan component.

The deconvolution of the N1s signal of HSAG-SP/CS aerogel and paper from a high-resolution XPS spectrum (Figure 10c) shows one dominant signal at 399.5 eV and one small contribution located at 401.5 eV. These signals correspond to free amine or amide groups of the chitosan chains (i.e., non-protonated nitrogen functionalities) and to protonated amines, respectively [24]. The dissolution process in acetic acid is in fact capable of inducing some protonation of the amine segments and few of these groups may remain protonated in the dry samples.

#### 3.5.4. WAXD Analysis

**Figure 11:**
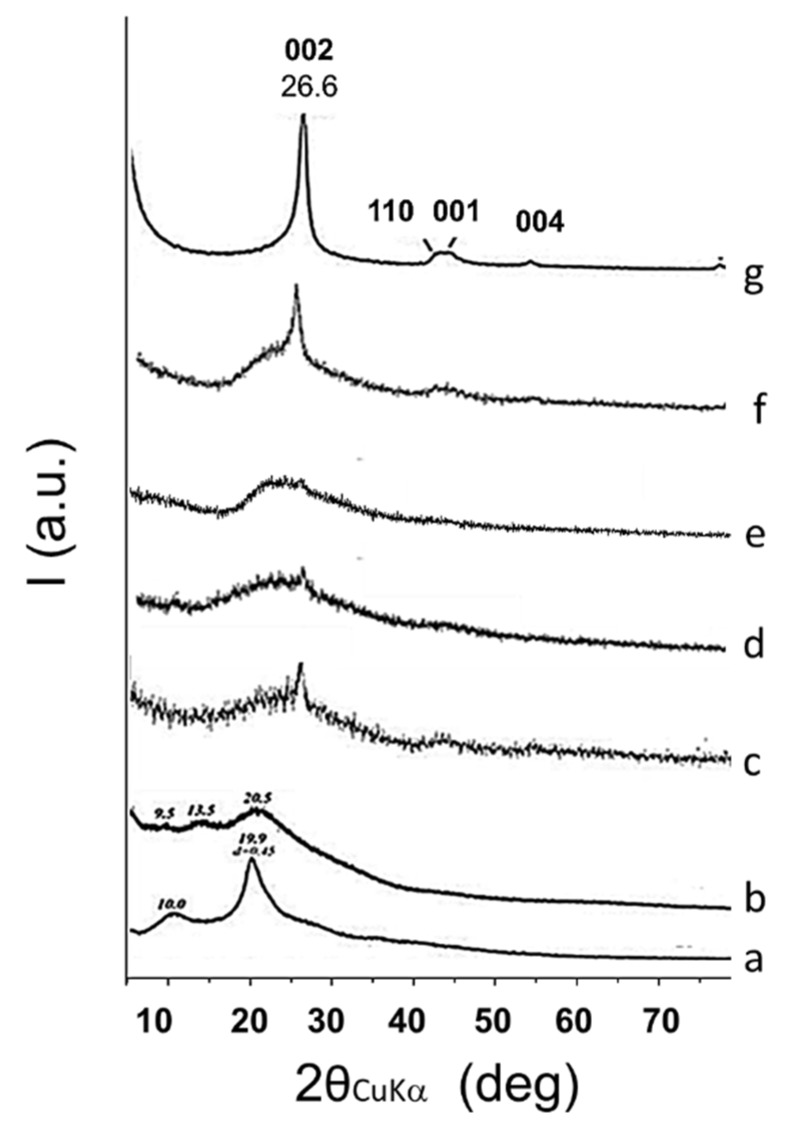
X-ray diffraction patterns (CuKα) of the CS powder (**a**), CS film in acetic acid (**b**), HSAG–SP/CS 1:1 paper (**c**), HSAG–SP/CS 1:1 aerogel (**d**), HSAG–SP/CS 1:1 aerogel (diluted) (**e**), HSAG/CS 1:1 aerogel (**f**) and HSAG–SP adduct (**g**).

Organization at the solid state of HSAG–SP/CS composites was investigated by means of WAXD analysis. Figure 11 shows WAXD patterns of CS powder (a), CS film in acetic acid (b), HSAG–SP/CS 1:1 paper (c), HSAG–SP/CS 1:1 aerogel (d and e), HSAG/CS 1:1 aerogel (f) and HSAG–SP adduct (g).

The two reflections at 10.0° and 19.9° as 2θ angles demonstrate the CS crystallinity (Figure 11a). The pattern of an isolated film of CS treated with acetic acid (in the absence of HSAG) in Figure 11b reveals crystalline peaks at 20.5° and 11.8° as the 2θ value. The WAXD pattern of HSAG–SP (Figure 11g) shows (*00ℓ*) reflections that indicate the order in the direction orthogonal to structural layers: (002) at 26.6°, which corresponds to an interlayer distance of 0.338 nm, and (004) at 54.3°. The in-plane order is proven by the (100) and (110) reflections at 42.5° and 77.6°, respectively. By using (002) reflection and applying the Scherrer equation (Equation (1)), the out of plane correlation length (*D*_┴_) was calculated and about 29 stacked layers were estimated. 

In the pattern of HSAG–SP/CS 1:1 carbon paper (Figure 11c), the (002) reflection remains at the same 2θ value as in the pattern of HSAG–SP. The peak shape analysis of (002) reflection allowed to evaluate the crystallite size, which was found to be appreciably reduced with respect to HSAG–SP. A number of about 10 layers stacked in the orthogonal direction with respect to basal planes was estimated. Such a reduction of graphene layers stacking could be attributed to the interaction between HSAG and CS, which is favored by the dispersion in water of HSAG functionalized with SP. Peaks due to crystalline chitosan could not be observed. Besides the reduction of CS mass in the analyzed sample (50% in the adduct), in the light of the pattern of CS treated with acetic acid shown in Figure 11b, it could be commented that the presence of HSAG inhibits the CS crystallization in the carbon paper. 

The pattern of the HSAG–SP/CS aerogel in Figure 11d is quite intriguing, since the (002) reflection is only slightly detectable. This finding indicates that exfoliation of HSAG–SP in the aerogel was promoted to a very large extent by CS treated with acetic acid, in spite of the large amount of HSAG in the sample (HSAG:CS = 1:1). It is indeed worth comparing the pattern of HSAG–SP/CS in Figure 11d with the pattern of HSAG/CS in Figure 11f. In the latter case, the (002) reflection appears clearly visible and pretty sharp. It can be thus commented that the exfoliation of nanosized graphitic aggregates is due to the synergy between CS and the functionalization of HSAG with SP. The small peak at 2θ = 8.4° can be attributed to the hydrated crystalline form of chitosan. In the literature, polymer crystallization induced by nanosized carbon allotropes, such as carbon nanotubes, is documented [25,26]. In the case of HSAG–SP/CS composites discussed in this paper, crystallinity can be observed only in the aerogel. 

**Figure 12:**
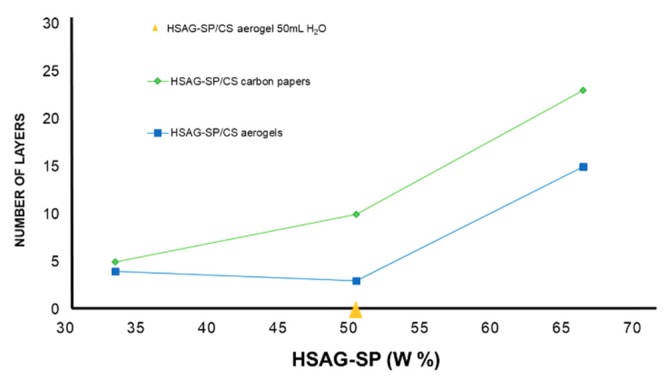
Number of stacked layers in graphitic aggregates in direction orthogonal to basal planes, evaluated from (002) peak shape analysis (Equation (1)), as a function of HSAG–SP mass% in the adduct with CS.

In the graph of Figure 12, the number of stacked graphene layers, evaluated by peak shape analysis of (002) reflection in WAXD spectra, is plotted as a function of the relative amount of HSAG–SP in the composite. 

As mentioned in the introduction, according to the ISO organization, well defined stacks consisting of three to ten layers, as those shown in Figure 4d and which gave rise to the WAXD pattern in Figure 11d, are named as few-layer graphene. Composites with few-layer graphene are thus obtained, particularly in aerogels, even in samples with 50 as mass % of the carbon material in the adduct. 

It is worth adding that X-ray investigation was performed also on HSAG–SP/CS aerogels having different ratios between the carbon material and CS: 1:4 and 4:1. The (002) reflection was observed in the latter sample but not in the composite with 20% by mass of HSAG–SP (1:4 HSAG–SP/CS). Such finding can be attributed to the pronounced exfoliation of HSAG but could also be due to its low relative amount (20% by mass). 

Water was added to the aerogel with 50% HSAG–SP (WAXD pattern in Figure 11e). The (002) reflection cannot be detected in the corresponding WAXD profile (the triangle in Figure 12 lies on the x axis). This finding indicates that the preparation of a composite in the form of aerogel based on the adduct between HSAG–SP and CS is indeed a suitable procedure to have highly exfoliated graphene layers, even in samples with large amount of carbon material. 

#### 3.5.5. SEM Analysis

**Figure 13:**
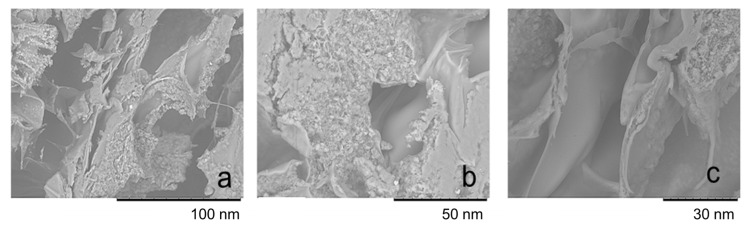
SEM micrographs of an HSAG–SP/CS 1:1 aerogel at different magnifications: 100 nm (**a**), 50 nm (**b**) and 30 nm (**c**).

Morphological properties of the aerogels were studied using scanning electron microscopy. Micrographs of the HSAG–SP/CS aerogel containing 50 mass % HSAG–SP (1:1 as the mass ratio) are shown in Figure 13.

Aerogels exhibit a spongy like structure. The highly porous structure is confirmed by the low density, equal to 0.026 g cm^−3^. The walls of the cavity are of chitosan, in the presence or in the absence of HSAG–SP, which is in most cases present in large aggregates. A continuous filler network is visible in some areas. This structure accounts for the electrical conductivity of the aerogel, discussed below in the text. 

#### 3.5.6. Electrical Conductivity of Aerogels and Carbon Papers Based on HSAG–SP and Chitosan

The four-point probe method was used to measure the electrical conductivity of HSAG–SP/CS aerogels [18]. Table 2 shows values of DC conductivity for aerogel and paper (discussed below in the text) with a different HSAG–SP/CS ratio. Measurements were repeated on several samples and the variation coefficient was found to be ≤3%.

A critical value of HSAG–SP/CS ratio exists for having values of electrical conductivity in line with those reported in the literature for carbon papers and aerogels. HSAG–SP/CS 1:1 aerogel shows conductivity compared to the paper with the same HSAG–SP/CS ratio. By increasing the quantity of graphite (4:1 aerogel) conductivity, as expected, increases (1.1 × 10^5^ μS/cm). 

Graphite and graphene have been considered to be outstanding candidates for supercapacitor fabrication due to their stable thermal and mechanical properties, high specific surface area, high electrical conductivity and stable chemical properties. In the literature, there are few reports on the preparation of chitosan paper and aerogel for supercapacitor electrode applications, but they are difficult to scale up and require the carbonization of chitosan to produce the carbon composite [24,25].

A very interesting perspective for carbon paper and aerogel prepared in the present work could be their application as electrodes in supercapacitors because of their low cost and environmentally friendly nature. However, the level of conductivity appears to be too low at present. To increase the electrical conductivity, different types of carbon allotropes, particularly of graphite, could be used, in the frame of the same CS-based experimental approach.

#### 3.5.7. Resistance to Solvents of Aerogels and Carbon Papers Based on HSAG–SP and Chitosan

Resistance of HSAG–SP/CS aerogels and carbon papers to solvents was preliminarily investigated. Vials containing specimens of the HSAG–SP/CS 1:1 sample were kept for two months in H_2_O (a), *n*-hexane (b) and DMF (c), as shown in Figure S3 and Figure S4 for carbon papers and aerogels, respectively. Swelling was not observed for carbon paper samples, recovered from the vials and TGA analysis did not reveal any mass loss that could be attributed to the sorbed solvent. In the case of aerogel samples, swelling was observed only in polar solvents, in particular in water. 

The stability of HSAG–SP/CS aerogels in a water solution having different pH was investigated, using substances such as HCl, CH_3_COOH, NaHCO_3_ and KOH to modulate pH values. Figure S5 shows the HSAG–SP/CS 1:1 aerogel dipped in water solutions at different pH values. In line with what shown in Figure S4, swelling was clearly detected at every pH value and was more pronounced at pH = 4 and 9. In a work available in the literature [27], a chitosan/CNT adduct was formed by adding CNT to an acidic chitosan solution (pH = 3.5–5) and the stability of the adduct up to a neutral pH was demonstrated. Thus, it could be hypothesized that the stability of the interaction between the polycation CS and the graphitic substrate is in a pretty wide range of pH. Such stability accounts for the swelling of the composite material, in which the chitosan chains are crosslinked by the graphene layers. 

## 4. Conclusions

This paper reports a novel and simple approach to achieve ultra-high exfoliation of graphitic aggregates: An adduct between edge functionalized graphene layers and cationic chitosan was formed in water and subjected to mild sonication. Few-layer graphene was obtained, and the structure of the layers was not appreciably perturbed. 

In previous reports it was shown that the sonication in water of either the same graphitic material (HSAG), edge functionalized with the same pyrrole compound [11], or of an adduct between cationic CS and the pristine HSAG [16] did not lead to high exfoliation. It seems that the edge functionalization and the interaction in acidic water with a cationic chitosan give rise to a synergic effect. 

Carbon papers and aerogels based on graphene layers and containing high amounts (up to 50% by mass) graphitic material were successfully prepared. Moreover, only few of the graphene layers were stacked or their exfoliation was so extensive that the X-ray pattern of the aerogel swollen in water did not reveal the typical (002) reflection of graphitic aggregates. 

The procedure here reported for obtaining few-layer graphene and highly exfoliated graphene in polymer matrices is sustainable in many aspects. A polymer, such as chitosan, was used. An HSAG–SP adduct was prepared with a high yield and thus almost no wastes (in the absence of organic solvents or catalysts), with the help of only thermal energy. HSAG–SP was mixed with chitosan in water, in the presence of only acetic acid, obtaining stable suspensions. Carbon papers and aerogels were obtained by simple casting and lyophilization of HSAG–SP/CS suspensions, respectively. 

Moreover, the nanocomposites were shown to have interesting properties: They were electrically conductive and stable for months with unaltered properties if stored in different solvents and at different pH.

## Figures and Tables

**Table 1:** Atomic percent concentration and concentration ratios deducted from XPS spectra of pristine HSAG–SP and of HSAG–SP/CS 1:1 aerogel and paper. The theoretical values calculated for pure chitosan are also reported.

	C1s (at. %)	O1s (at. %)	N1s (at. %)	O1s/C1s	N1s/O1s	N1s/C1s
HSAG–SP	91.5	6.5	2.0	0.07	0.31	0.02
HSAG–SP/CS aerogel	63	31	6.0	0.49	0.19	0.10
HSAG–SP/CS paper	65.1	28.8	6.1	0.44	0.21	0.09
CS theoretical	56.5	35.9	7.6	0.64	0.21	0.14

**Table 2:** Conductivity of HSAG–SP/CS composites with different HSAG–SP ratios.

HSAG–SP/CS Ratio ^a^	σ (μS/cm)
CS powder	1 × 10^−4^
CS paper	1 × 10^−4^
1:1 aerogel	14
1:1 aerogel ^b^	140
4:1 aerogel	1.1 × 10^5^
1:1 paper	10

^a^ Content with respect to 100 mg of chitosan; ^b^ after pyrolysis.

## Supplementary Materials

The following are available online at https://www.mdpi.com/1996-1944/13/1/39/s1, Figure S1: ^1^H NMR spectrum (DMSO-d6, 400 MHz) of 2-(2,5-dimethyl-1*H*-pyrrol-1-yl)-1,3-propanediol (SP), Figure S2: TGA traces of HSAG-SP (a), HSAG-SP/CS (1:1 as mass ratio) aerogel (b), and CS (c), Figure S3: HSAG/CS 1:1 carbon paper after 2 months storage in H_2_O (a), *n*-hexane (b) and DMF (c), Figure S4: HSAG-SP/CS 1:1 aerogel after 2 months storage in H_2_O (a), *n*-hexane (b) and DMF (c), Figure S5: HSAG-SP/CS 1:1 aerogel in water solutions having different pH.
