# Supplementary material for: Edge Functionalized Graphene Layers for (Ultra) High Exfoliation in Carbon Papers and Aerogels in the Presence of Chitosan
Source: Materials (Basel). 2019 Dec 20;13(1):39. doi: 10.3390/ma13010039 (PMC6981762; doi:10.3390/ma13010039)
Supplement: Supplementary file 1 [file materials-13-00039-s001.pdf]

materials

Supplymentary Materials

Edge Functionalized Graphene Layers for (Ultra) High Exfoliation in Carbon Papers and Aerogels in the Presence of Chitosan

Silvia Guerra [1], Vincenzina Barbera[1,*], Alessandra Vitale [2], Roberta Bongiovanni [2], Andrea Serafini [1], Lucia Conzatti [3], Luigi Brambilla [1] and Maurizio Galimberti [1,*]

[1] Politecnico di Milano, Department of Chemistry, Materials and Chemical Engineering "G. Natta", Via Mancinelli 7, 20131 Milan, Italy; silvia.guerra@polimi.it (S.G.); andrea.serafini@polimi.it (A.S.); luigi.brambilla@polimi.it (L.B.)

[2] Politecnico di Torino, Department of Applied Science and Technology, Corso Duca degli Abruzzi 24, 10129 Torino, Italy; alessandra.vitale@polito.it (A.V.); roberta.bongiovanni@polito.it (R.B.)

[3] Istituto di Scienze e Tecnologie Chimiche (SCITEC) "Giulio Natta", Via De Marini 6 – 16149 Genova, Italy; lucia.conzatti@ge.ismac.cnr.it

* Correspondence: vincenzina.barbera@polimi.it (V.B.); maurizio.galimberti@polimi.it (M.G.)

Received: 6 November 2019; Accepted: 12 December 2019; Published: date

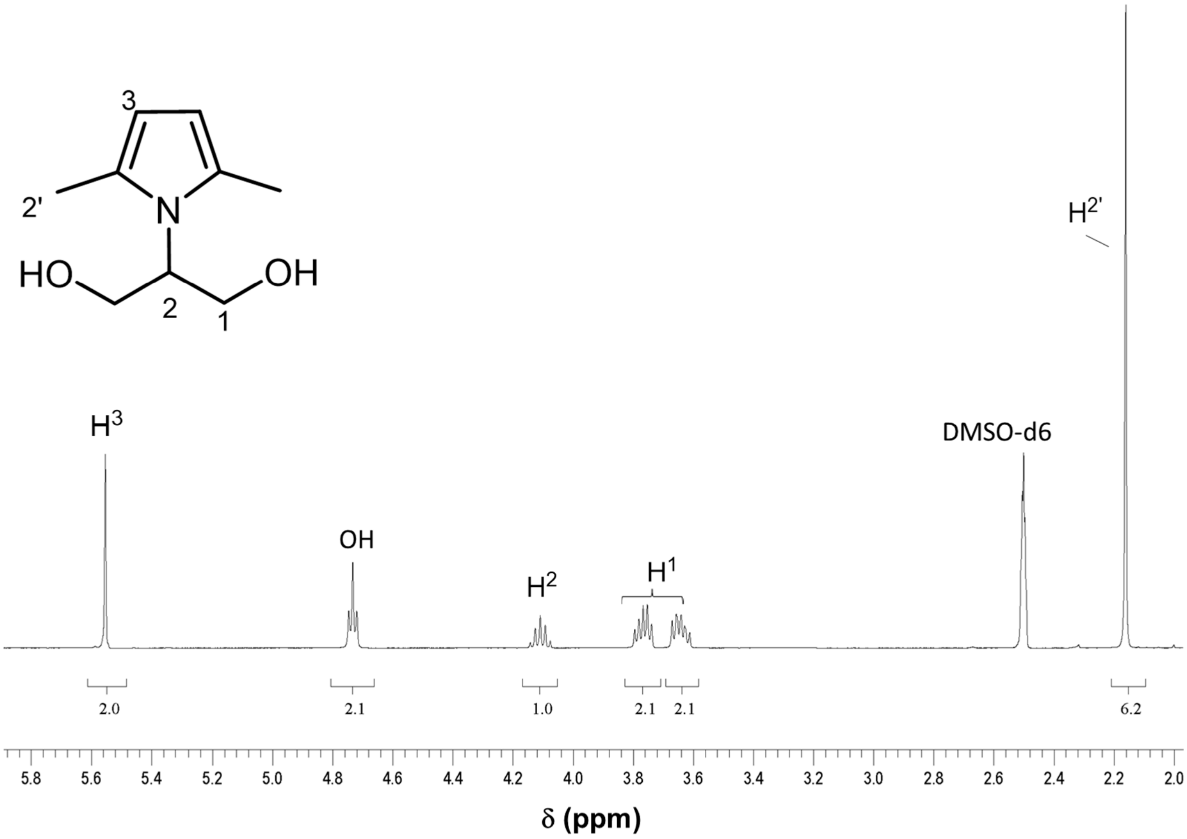

Figure S1. ^{1}H NMR spectrum (DMSO-d6, 400 MHz) of 2-(2,5-dimethyl-1*H*-pyrrol-1-yl)-1,3-propanediol (SP).

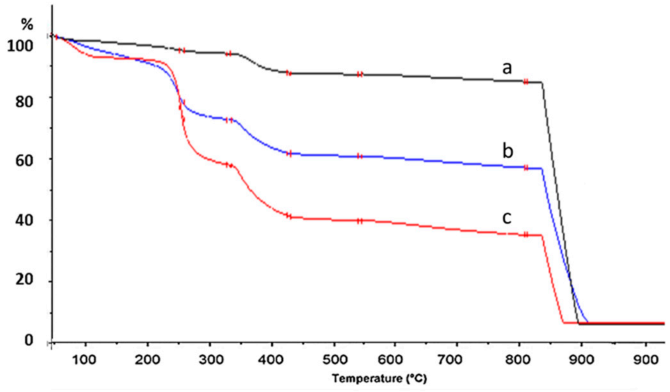

Figure S2. TGA traces of HSAG-SP (**a**), HSAG-SP/CS (1:1 as mass ratio) aerogel (**b**), and CS (**c**).

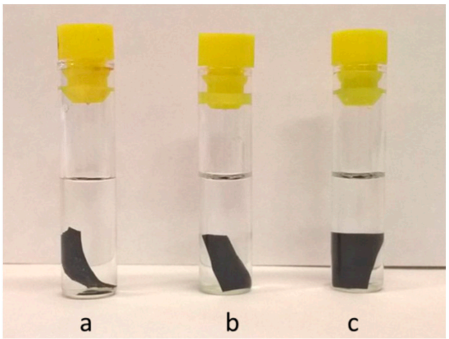

Figure S3. HSAG/CS 1:1 carbon paper after 2 months storage in H_2O (**a**), *n*-hexane (**b**) and DMF (**c**).

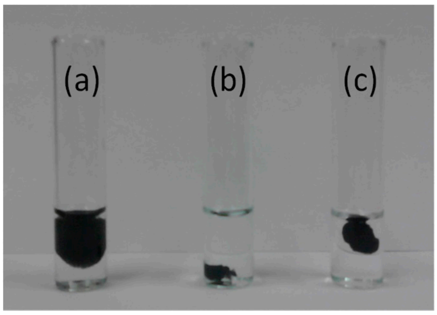

Figure S4. HSAG-SP/CS 1:1 aerogel after 2 months storage in H_2O (**a**) *n*-hexane (**b**) and DMF (**c**).

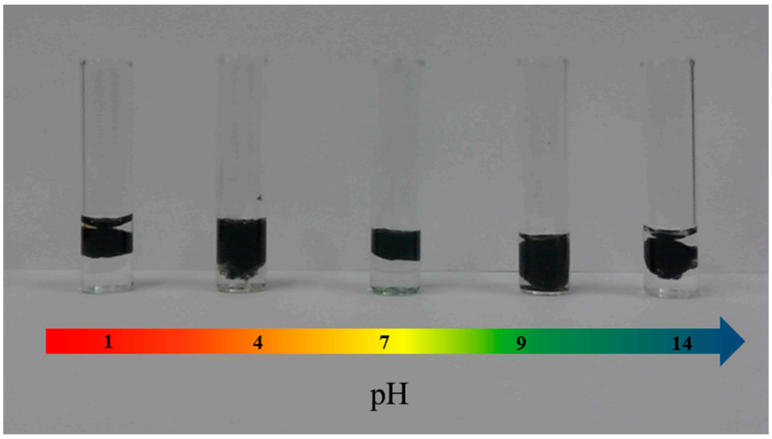

Figure S5. HSAG-SP/CS 1:1 aerogel in water solutions having different pH.

© 2019 by the authors. Submitted for possible open access publication under the terms and conditions of the Creative Commons Attribution (CC BY) license (http://creativecommons.org/licenses/by/4.0/).
